# Identifying the Functional Flexion-extension Axis of the Knee: An *In-Vivo* Kinematics Study

**DOI:** 10.1371/journal.pone.0128877

**Published:** 2015-06-03

**Authors:** Li Yin, Kaining Chen, Lin Guo, Liangjun Cheng, Fuyou Wang, Liu Yang

**Affiliations:** 1 Center for Joint Surgery, Southwest Hospital, the Third Military Medical University, Chongqing, People’s Republic of China; 2 Department of Radiology, Southwest Hospital, the Third Military Medical University, Chongqing, People’s Republic of China; University of Manchester, UNITED KINGDOM

## Abstract

**Purpose:**

This study aimed to calculate the flexion-extension axis (FEA) of the knee through in-vivo knee kinematics data, and then compare it with two major anatomical axes of the femoral condyles: the transepicondylar axis (TEA) defined by connecting the medial sulcus and lateral prominence, and the cylinder axis (CA) defined by connecting the centers of posterior condyles.

**Methods:**

The knee kinematics data of 20 healthy subjects were acquired under weight-bearing condition using bi-planar x-ray imaging and 3D-2D registration techniques. By tracking the vertical coordinate change of all points on the surface of femur during knee flexion, the FEA was determined as the line connecting the points with the least vertical shift in the medial and lateral condyles respectively. Angular deviation and distance among the TEA, CA and FEA were measured.

**Results:**

The TEA-FEA angular deviation was significantly larger than that of the CA-FEA in 3D and transverse plane (3.45° vs. 1.98°, p < 0.001; 2.72° vs. 1.19°, p = 0.002), but not in the coronal plane (1.61° vs. 0.83°, p = 0.076). The TEA-FEA distance was significantly greater than that of the CA-FEA in the medial side (6.7 mm vs. 1.9 mm, p < 0.001), but not in the lateral side (3.2 mm vs. 2.0 mm, p = 0.16).

**Conclusion:**

The CA is closer to the FEA compared with the TEA; it can better serve as an anatomical surrogate for the functional knee axis.

## Introduction

The kinematics of the knee joint has been extensively studied over the past decades. From early “instant centers” to later “finite helical axis”, the conventional concept is that there is no fixed axis for the flexion-extension movements to occur about, instead, an axis with changing position and orientation guides the motion [1, 2]. Although these models can precisely depict the knee kinematics in 3D space and 2D plane, the mathematical definition of the changing axis is complex and thus makes it hard for clinicians to understand. Later studies have suggested that the knee motion can be better described as simultaneous rotation about two fixed axes: the flexion-extension axis (FEA) in the posterior femoral condyles and the longitudinal axis (LRA) in the tibia [3, 4]. The advantages of this approach are that it simplifies the description of knee movements by decomposing the motion into two major components and associates the functional axis with certain anatomical landmarks. This facilitates researchers and clinicians to understand the knowledge and apply it in practice.

The association between the FEA and anatomical landmarks is of great interest to several clinical practices, such as total knee arthroplasty (TKA), prosthesis design and ligamental reconstruction. For example, the femoral components of contemporary TKA prosthesis are commonly featured with symmetrical condyles with identical sagittal curvature, the center of which should be ideally aligned with the FEA; during surgery, the alignment of components often relies on anatomical landmarks as references [5]. However, this function-to-anatomy relationship remains in dispute. Early studies based on in-vitro cadaver test have indicated that the transepicondylar axis (TEA) connecting the sulcus on the medial femoral condyle and the eminence on the lateral femoral condyle, served as a good anatomical surrogate for the FEA [3, 4, 6]. This notion was challenged recently by the in-vivo tracking of the TEA during functional knee movements [7]. In this study, the medial end of the TEA was observed to shift superiorly before 100° of knee flexion and move inferiorly thereafter, whereas the lateral end demonstrated nearly consistent inferior displacement throughout the range of flexion. These findings indicated that the TEA changed its orientation during knee motion and thus might not be the functional flexion-extension axis. In another study, notable angular deviation between the TEA and FEA was also observed [8]. Based on anatomical and geometrical measurements, some authors suggested that the cylinder axis (CA), which was the co-axis of two cylinders fitted to the medial and lateral posterior condyles of femur could better represent the FEA [9–11]. To clarify the location of the FEA and its association with anatomical landmarks, more kinematics studies, especially those under active weight-bearing conditions are still needed.

Moreover, there is one inherent drawback in the existing methods to calculate the FEA: the LRA ought to be calculated independently through the repeated movements of internal-external rotation of the knee prior to the FEA [3, 4, 12]. These procedures are achievable in in-vitro cadaver tests, but become less practical in studies collecting in-vivo kinematics during functional tasks, such as those employing open-access MRI and fluoroscopy. Therefore, developing a new method to calculate the FEA would be beneficial.

The aim of this study was to calculate the FEA based on the kinematics data of weight-bearing knee bending, and then associate it with two major anatomical axes defined in distal femur: the TEA and CA. We hypothesized that the CA was located more closely to the FEA than the TEA, by the angular deviation and the distance between the end points on the surfaces of femoral condyles.

## Methods

### Subject

Twenty healthy volunteers (16 males, 4 females) with the average age of 29.4 (SD 5.6, range 21–42) were recruited in this study. Each subject went through physical examination in the lower limbs before enrollment. Subjects with pain, deformity, injury or surgery history in either lower limb were excluded. For each subject, unilateral knee was randomly selected to participate in the following tests (10 left and 10 right in total). The study protocol was reviewed and approved by the ethic committee in the author’s institute. All subjects provided informed consent before participation.

### Knee modeling and kinematics data collection

A computer tomography (CT) scan was made for each involved knee on a 16-slice spiral scanner (SOMATOM, Siemens, Germany), covering a range of 30 cm centered on the joint line. The images were acquired with the slice thickness of 1 mm and the in-plane resolution of 512*512. Before scanning, the mechanical axis of the involved lower limb was measured and aligned with the longitudinal direction of the examining bed. The image data was segmented and reconstructed into 3D models by medical image processing software (Mimics 14.1, Materialise, Belgium).

The bi-planar X-ray imaging and 3D-2D registration techniques were used to acquire the in-vivo kinematics data. The bi-planar imaging system was customized by a fixed digital radiographing (DR) system and a mobile DR system, which were set up to make the beams cross orthogonally. The subjects performed single-leg lunge maneuvers at 0°, 15°, 30°, 60°, 90°, 120° of knee flexion in the imaging area. At each flexion angle, two radiographs were taken simultaneously. The knee flexion angles were repeatedly measured prior to the image capture, and later verified in the radiographs. The maximal tolerable error was 3°.

A virtual environment was later built according to the physical positions of the two DR systems. The 3D knee models were registered manually in the virtual environment by matching their projected shapes to both of the radiographs. The registered models of the six knee flexion angles were then integrated, by which the successive in-vivo tibiofemoral movements were digitally restored. The translational and rotational error brought by the 3D-2D registration techniques have been reported to be less than 0.5 mm and 0.5° [6, 13]; similar accuracy has also been validated in our previous study [14].

### Mathematical analysis

A knee coordinate system was built in tibia according to Grood and Suntay’s description [15]. The origin was located at the center of the tibial plateau; the Z axis was parallel with the longitudinal axis of tibia, directing proximally; the X axis was in the plane perpendicular to the Z axis (transverse plane), directing right and passing through the centers of the medial and lateral tibial plateau projected onto the transverse plane; the Y axis was the cross-product of the X and Z axes, directing anteriorly. The centers of the medial and lateral tibial plateau were located using circle fit of the bony contours, which has been described by Cobb and Victor [16, 17]. This method has been demonstrated to have high precision and good inter-observer reliability [16].

**Fig 1:**
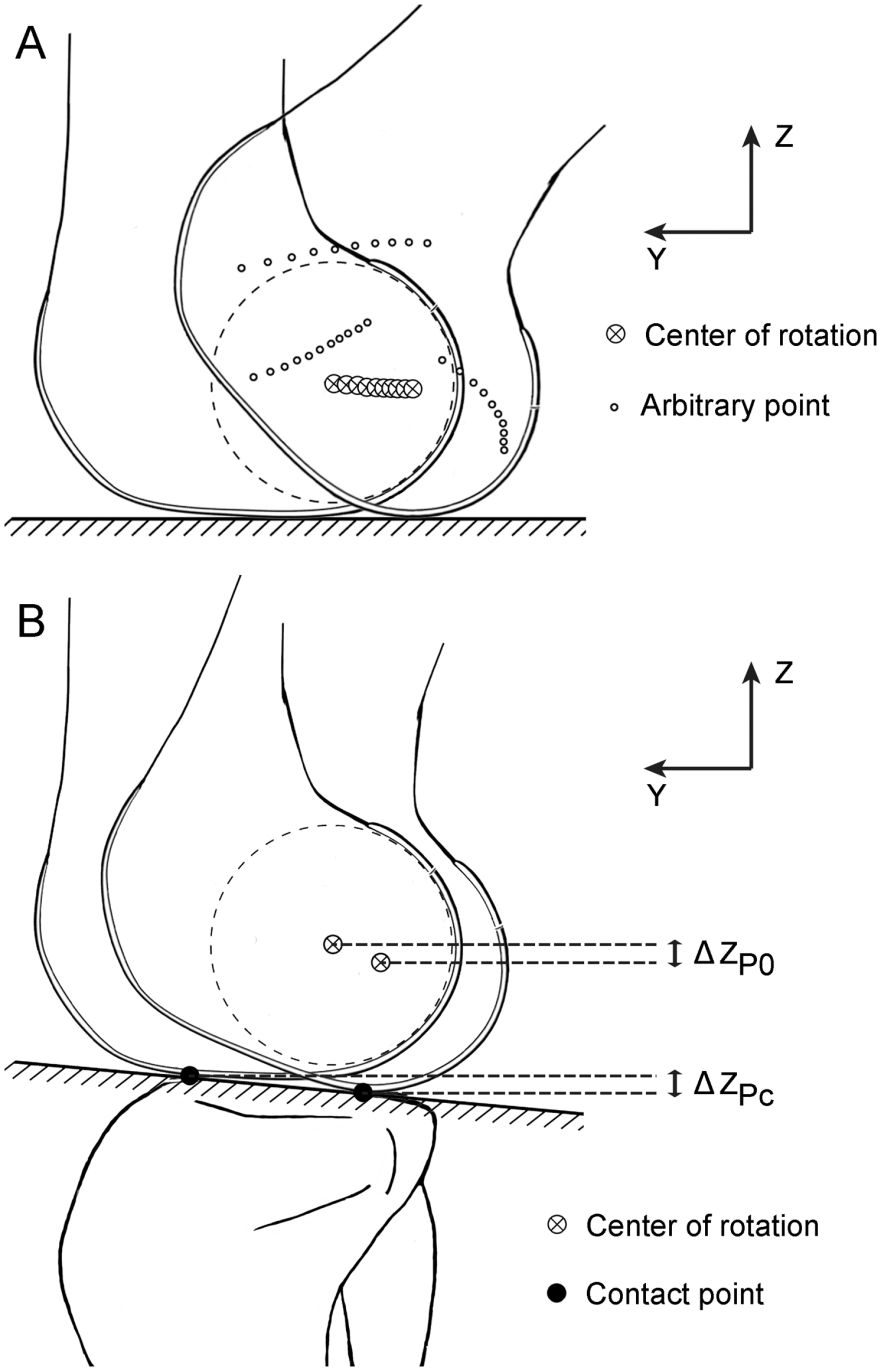
The algorithm to calculate the center of rotation of the femoral condyle. A. The center of rotation in a rolling object with circular contact profile keeps a constant vertical distance to the supporting ground; points away from the center of rotation undergo vertical shifts of different magnitudes, depended on their respective distance to the center of rotation. B. When the rolling occurs on an oblique ground, the center of rotation declines naturally; the amount of downward shift of the center of rotation (ΔZ_p0_) approximates that of the contact points (ΔZ_p*c*_).

The algorithm of calculating the FEA from the consecutive models of knee bending is based on the fact that, the center of rotation remains a relatively steady vertical distance to the tibia plateau, regardless of the motional patterns of femoral condyles (sliding, rolling or both). An intuitive analogy to this is a running car, of which the roll centers always keep a constant height to the ground. From this point of view, out of all of the geometric points in the femur the center of rotation would have the least amount of vertical shift throughout the knee flexion-extension, which approximates zero (Fig 1A).

Since the longitudinal axis of tibia has been defined as the Z axis in the knee coordinate system, for any point *P*_*n*_ in the femur the vertical shift value (VSV) in relation to the tibial plateau during a single flexion interval *i* can be written as:

$${\text{VSV}}_{\text{Pn},\text{i}}=\left|{\Delta \text{Z}}_{\text{Pn},\text{i}}\right|$$

The cumulative vertical shift value (cVSV) of the flexion intervals 1 through k is the sum of the VSV of each individual flexion interval,

$${\text{cVSV}}_{\text{Pn}}=\overset{\text{k}}{\underset{\text{i}=1}{\sum }}\left|{\Delta \text{Z}}_{\text{Pn},\text{i}}\right|$$

If *P*_0_ is the center of rotation, it should have the least cVSV of all points.

$${\text{cVSV}}_{\text{P}0}=\text{min}\{{\text{cVSV}}_{\text{P}0},{\text{cVSV}}_{\text{P}1},{\text{ cVSV}}_{\text{P}2},{\text{ cVSV}}_{\text{P}3}\ldots \ldots {\text{cVSV}}_{\text{Pn}}\}$$

Due to the existence of tibial slope in the sagittal plane, the vertical distance of the femoral condyles to the tibial plateau will naturally decrease as long as “roll back” happens. Therefore, the VSV and cVSV were compensated by the vertical shift of the tibiofemoral contact points, which represented the movements of the femur as a whole in the vertical direction. Specifically, the contact points at each flexion angle were located by finding the shortest distance between the tibial plateau and the surfaces of femoral condyles and then defined as the midpoint of the line crossing this distance; the VSV was compensated by subtracting the vertical shift of the contact points (Fig 1B). If *P*_*c*_ is the contact point, the compensation can be written as:

$${\text{VSV}}_{\text{Pn},\text{i }}=\left|{\Delta \text{Z}}_{\text{Pn},\text{i}}-{\Delta \text{Z}}_{\text{Pc},\text{i}}\right|$$

The geometric model of femur was discretized to point cloud with the average point-to-point distance of 0.1 mm. To reduce computing time, only those points on the femoral condyles were manually selected for further tests. The number of the selected points approximated 50,000 in each model. The spatial coordinates of the selected points were tracked in the six flexion phase (0°, 15°, 30°, 60°, 90°, 120°) and the cVSV of the five corresponding flexion intervals was calculated. All of the computations mentioned above were accomplished using a custom-written script in an open-source numerical computational package (Scilab, Scilab Enterprises, France). The end points of the FEA were thus defined as the points with the least cVSV screened out from the point cloud.

### Measurements and statistics

**Fig 2:**
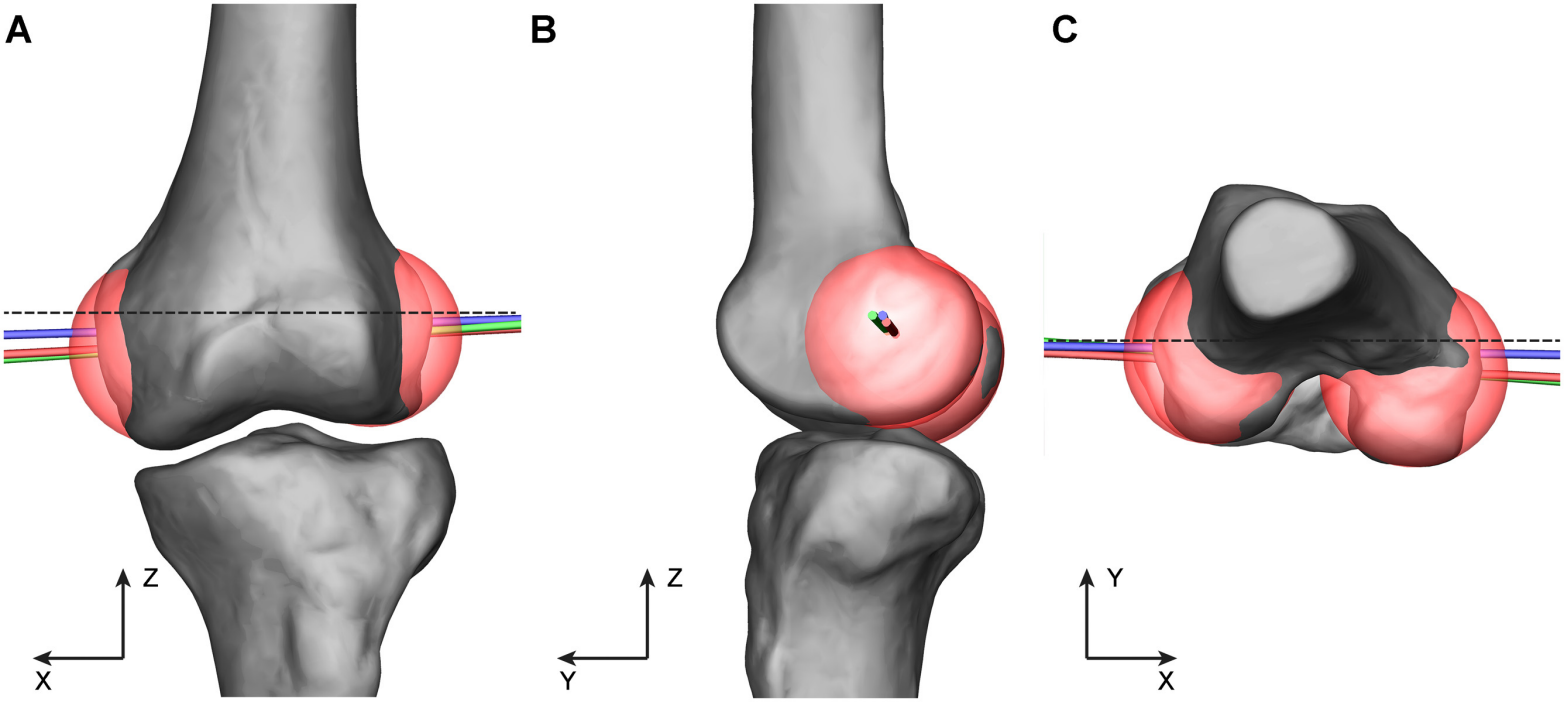
The positions and orientations of the transepicondylar axis (TEA), cylinder axis (CA) and the computed flexion-extension axis (FEA). The TEA (marked blue) is defined by connecting the most prominent point on the lateral epicondyle and the sulcus point (or, when absent, the prominence) on the medial epicondyle; The CA (marked red) is defined by connecting the two centers of the best fitted spheres to the medial and lateral posterior condyles. The FEA is marked green. The absolute angles of the three axes in the coronal and transverse plane are measured by their in-plane projections in relation to the X axis. The dashed line represents the orientation of the X axis. A. Coronal view. B. Sagittal view. C. Transverse view.

The FEA was compared with two major anatomical reference axes of the femoral condyles: the TEA and CA. In our study, the TEA was visually identified by connecting the most prominent point on the lateral epicondyle and the sulcus point (or, when absent, the prominence) on the medial epicondyle of the 3D model based on the method described by Most et al. [18]. The CA was defined by connecting the two centers of the best fitted spheres to the medial and lateral posterior condyles in a least square sense, which was similar to the definition of the so-called geometric center axis in previous studies [18–21]. We found that this method led to identical results but was easier and more prevailing than conducting a cylinder fit to both condyles. After the CA was defined, it was elongated to intersect the surfaces of femoral condyles; the intersections were defined as the end points of the CA (Fig 2).

The absolute angles of the FEA, TEA and CA were first measured in the coronal and transverse plane by their projections in relation to the X axis of the knee coordinate system (Fig 2). The in-plane angular deviations between the FEA and TEA and between the FEA and CA were then quantified respectively. The 3D angles among these three axes were calculated using the dot product, which described the angle in 3D space between two lines that do not intersect [10]. In addition, the distances of the end points on the condylar surfaces of these three axes were measured and compared. All datasets were checked for normal distribution. One-way repeated measure ANOVA was conducted to compare the absolute angles among the FEA, TEA and CA in the coronal and transverse plane; Bonferroni procedure was used in post hoc tests. Paired sample t-tests were performed to examine the angular deviation as well as the distance of the end points between the FEA and TEA and between the FEA and CA to compare their closeness. Statistical significance level was set at 0.05.

## Results

**Fig 3:**
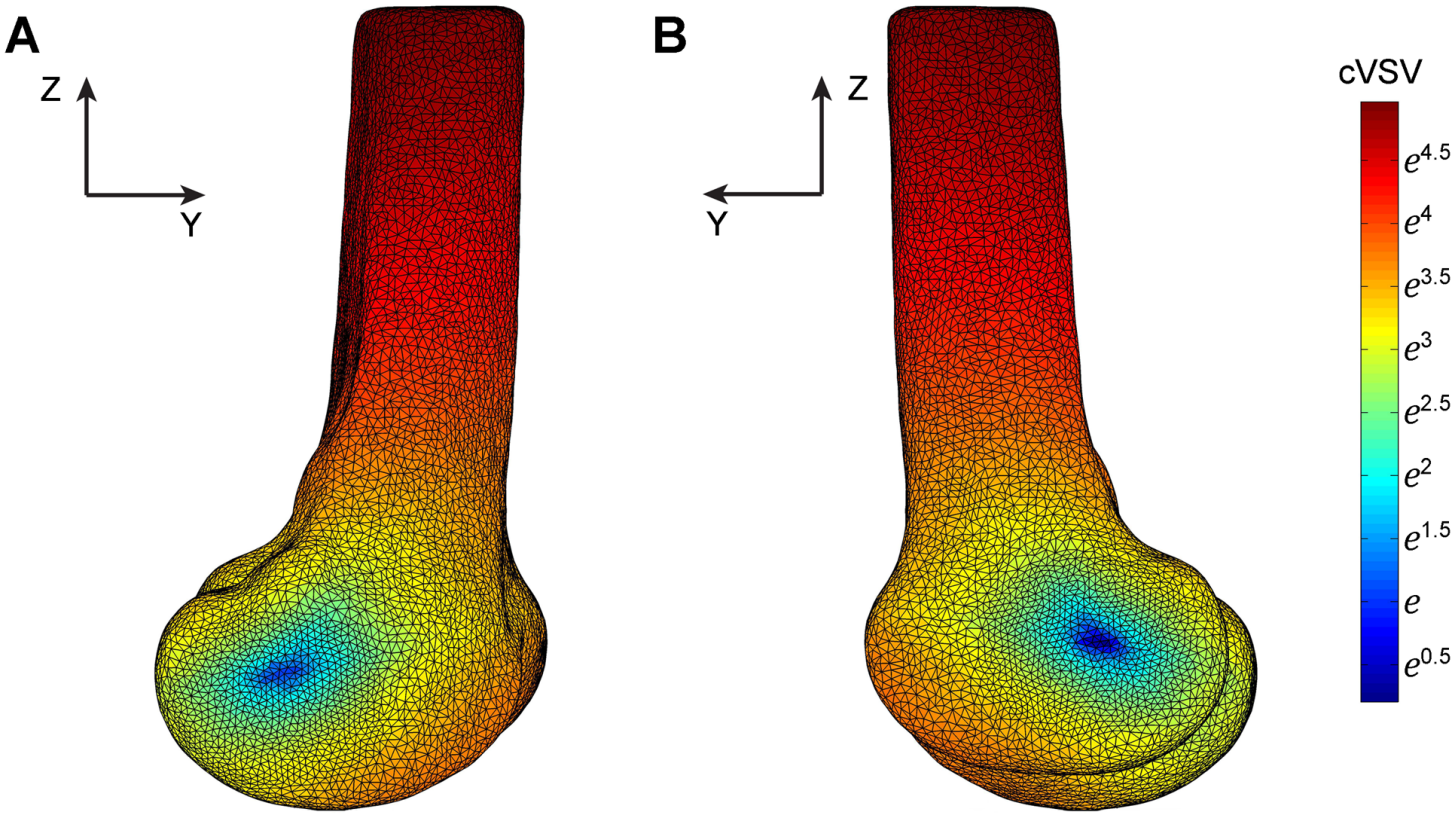
The mapping of the cumulative vertical shift value (cVSV). The graph shows the distribution of the cVSV from 0° to 120° of knee flexion in the left femur of one subject. The points with the lowest cVSV concentrate at the center of posterior condyles. A. Medial view. B. Lateral view.

The points with low cVSV concentrated at the central portion of the medial and lateral posterior condyles, where the end points of the FEA were selected (Fig 3). In the coronal plane, the absolute angle of the FEA was significantly different from that of TEA (P < 0.001), but not from the CA (P = 0.065); in the transverse plane, both the TEA and CA were found significantly different from the FEA (P < 0.001, P = 0.007, respectively) (Table 1). The CA, when compared with the TEA, showed overall lower angular deviation with the FEA in 3D and planes. The difference between the angular deviation of TEA-FEA and CA-FEA was significant in 3D and transverse plane, but not in the coronal plane (Table 2).

**Table 1:** The absolute angles of the FEA, TEA and CA in the coronal and the transverse plane.

	FEA				TEA				CA			
	Mean	SD	Min	Max	Mean	SD	Min	Max	Mean	SD	Min	Max
Coronal	3.99	1.73	1.17	6.44	2.38	2.01	-2.64	6.75	3.16	1.66	-0.55	5.21
Transverse	-8.04	4.61	-15.69	0.45	-5.32	4.70	-13.18	1.71	-6.85	4.46	-15.20	0.99

Angles are measured with respect to the X axis of the knee coordinate system built on tibia. All values are expressed in degrees. Relative abduction in the coronal plane and relative external rotation in the transverse plane are marked as positive values.

**Table 2:** The angular deviation between the TEA and FEA, and between the CA and FEA.

	TEA-FEA				CA-FEA				
	Mean	SD	Min	Max	Mean	SD	Min	Max	P
3D	3.45	1.58	0.27	6.36	1.98	1.55	0.42	6.25	<0.001
Coronal	-1.61	1.67	-5.04	0.76	-0.83	0.98	-3.05	1.21	0.076
Transverse	2.72	1.34	0.27	5.07	1.19	1.85	-1.27	5.54	0.002

All values are expressed in degrees. Relative abduction in the frontal plane and relative external rotation in the transverse plane are marked as positive values.

**Fig 4:**
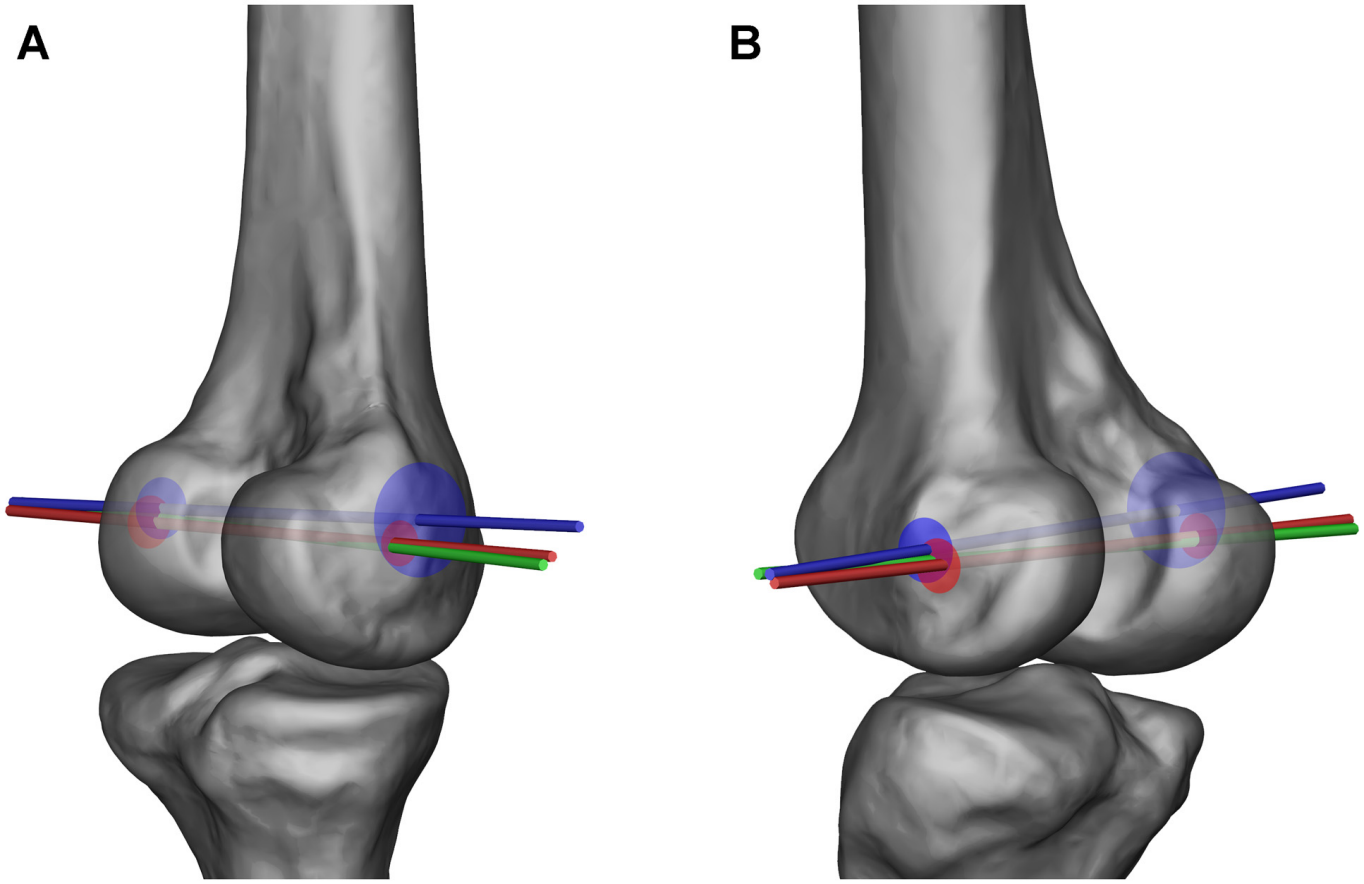
The positional relationship between the flexion-extension axis (FEA) and two anatomical axes. The FEA is marked green, the transepicondylar axis (TEA) is marked blue, and the cylinder axis (CA) is marked red. The presented positions of the CA and TEA were identified by averaging the locations of their end points related to those of the FEA throughout the 20 subjects. The red and blue circles represent the 95% confidence interval of the positions of the CA and TEA end points, respectively. A. Posteromeidal view. B. Posterolateral view.

The distance between the end points of the FEA and TEA averaged 6.7 mm (SD 2.2 mm, range 3.4–9.4 mm) on the medial side and 3.2 mm (SD 1.5 mm, range 1.7–6.7 mm) on the lateral side. The distance between the end points of the FEA and CA averaged 1.9 mm (SD 1.5 mm, range 0.1–5.4 mm) on the medial side and 2.0 mm (SD 1.6 mm, range 0.2–6.6 mm) on the lateral side. Significant difference was found between the deviation of TEA-FEA and CA-FEA on the medial side (P < 0.001); but not on the lateral side (P = 0.16). According to the positions of the end points, the TEA was found superior and anterior to the FEA in general, while no preferential relationship was found between the CA and FEA (Fig 4).

## Discussion

The main finding in our study was that neither the TEA nor the CA could perfectly coincide with functional flexion-extension axis of the knee; however, the CA exhibited better approximation to the latter than the TEA did. This was confirmed by their angular deviation and distance between end points, which also proved our hypotheses. The relatively small discrepancy between the CA and the FEA indicated that it could serve as a better anatomical surrogate than the TEA when the FEA could not be directly measured. Our findings were well supported by previous anatomical studies. Elias et al [22] found that the posterior condyles of femur appeared circular and were superimposed when viewed along the line across the origins of collateral ligaments, indicating the femur flexes about a fixed axis connecting the condyle centers. Pinskerova et al [23] suggested that the shape of the femoral condyles were composed of the arcs of two circles: the short extension arc and the long flexion arc; for most of the flexion period, the femur rotated about the center of flexion arc. These mentioned rotational center and axis were consistent with the CA in our study.

The locations of the FEA and its corresponding anatomical landmarks have been differently documented in previous literatures. In an in-vitro cadaver study, Hollister et al [4] found that the FEA ran through the origins of the medial and lateral collateral ligaments; and when it was viewed end-on, the femoral condyles were superimposed and appeared circular shapes. In another cadaver loading test, Churchill et al [3] concluded that the TEA approximated the FEA with an angular deviation of 2.9° (SD 1.2°). In an in-vivo study, Asano et al [6] observed no significant difference between the medial and lateral end points of TEA and FEA when measured in CT images, suggesting that the TEA well approximated the FEA. Converse conclusion was reached in a recent study by Mochizuki et al [7]. They tracked the position of TEA during in-vivo knee flexion, and found that the movements of its two end points in the vertical direction were prominent and inconsistent, suggesting that the TEA was offset from the true axis of rotation. Through morphologic analysis of the lower limb and local knee, Eckhoff et al [9, 10] suggested that the CA coincided with the FEA, while the angular deviation between the TEA and FEA existed in the 3D space (4.6°), coronal plane (1.8°) and transverse plane (2.3°). A possible reason for these different results could be, although discrepancy existed among the FEA, TEA and CA, it was relatively small with regard to the physical scale of knee, and could be further suppressed when these axes were projected to planes; some study revealed that the deviation between the TEA and CA could be as small as 0.21° (SD 1.77°) on the transverse plane [17]. Therefore this discrepancy was likely to be ignored under some circumstances. In these studies which advocated that the TEA was a better surrogate than the CA [3, 6], the author also reported superimposed and circularly shaped femoral condyles when the view was set along with the FEA. In addition, the notable inter-individual variation and inter-observer error of the TEA may also contribute to the differing results [24, 25].

The algorithm we used to analyze the knee motion and calculate the FEA was based on a simple rule of rigid body rolling, that the center of rotation maintained a relatively stable height to the supporting ground. This algorithm featured that it was independent of the motional pattern of the femoral condyles (rolling, spinning or sliding), and would not be affected by the transverse movements of the knee during flexion (e.g. internal rotation of tibia). Traditional methods calculate the FEA by tracking markers fixed on the tibia, which is vulnerably affected by transverse movements of the knee. To eliminate these disturbances, previous studies first located the LRA, about which the intrinsic transverse rotation of tibia happened; upon its location, the FEA would be calculated by the trajectories of the markers on the LRA, which tended to stay in plane and exhibit concentric circles [3, 4, 12]. This method required extra test steps of repeated internal-external rotation being applied to the tibia at various knee flexion angles. Although it has been well performed in in-vitro knee rig simulations, it could be hard to implement in in-vivo studies, such as those using open MRI, image matching and Roentgen stereophotogrammetric analysis (RSA). For this reason, some authors used the estimated LRA to calculate the FEA in in-vivo study [6], by which the accuracy of the dependent FEA may be affected. In addition, the transverse movements of the knee can be essentially influenced by various external factors, such as the knee loading pattern, muscle activity, positions of the feet and so on [26–28]. This indicates that the LRA may not be identical across different maneuver tasks, which may induce extra errors to the calculated FEA. Therefore, our method may be more suitable for those in-vivo and multi-activity studies.

Both CA and TEA have been used in the biomechanical and clinical studies of the knee. In kinematics studies, the knee motions are usually described as the transverse movements of the femoral condyles represented by a single axis as a function of knee flexion [29, 30]. As evaluation parameters, the CA and TEA have been demonstrated to lead to divergent results [18, 19]. Employing an axis close to the FEA can maximally eliminate the error caused by flexion thus reveal the true transverse movements. From this point of view, the CA may be a superior choice. We advocate using the anatomical reference frame suggested by Victor et al [17], which is established based on the CA to study and describe the knee kinematics. In TKA, the TEA traditionally serves as a major reference line to align femoral components. However, the revealed angular deviation implies that it may contribute to the midrange instability in those “single-radius” prostheses which are designed to be kinematically aligned to the FEA [7, 10]. As has been proven to approximate the FEA, the CA has also been demonstrated to be more perpendicular to the mechanical axis of the tibia as well as the entire lower limb compared with the TEA [11, 17]. Therefore, aligning these prostheses according to the CA may better replicate the normal knee kinematics, as has been advocated by some authors [10, 11]. However, more biomechanical and especially clinical studies are still needed to support this conclusion. Current TKA surgery requires removing bone from the lateral tibia and medial femur to keep symmetrical extension and flexion joint spaces as well as balanced soft tissue, which may change the flexion-extension axis of the post-operative knee [5]. Restoring the physiological knee flexion pattern does not only rely on surgical techniques, but also needs incorporated effort from biomechanical researchers and prosthesis designers.

There are still several limitations and challenges in previous and current studies seeking the FEA and its anatomical surrogates. The number of subjects were usually small (under 25) and sampled from a single population across studies [3, 8, 10, 17]. Knee kinematics was collected using various methods, such as cadaver tests using knee simulator [3], optical tracking using surgery navigation system [8], in-vivo radiographic tracking based on single-planar or bi-planar image matching techniques [6, 7]; meanwhile, the kinematics was under different loading conditions, such as passive flexion [4], quasi-static knee bending (current study) and gait [13]. All these factors may put an impact on the results, which need to be future clarified.

## Conclusion

Compared with the TEA, the CA is closer to the functional flexion-extension axis of the knee. The CA can serve as a better reference axis to evaluate and describe the knee kinematics, and also has the potentialities to guide the component alignment in TKA. More future studies are needed to clarify its clinical importance and usefulness.
